# Decreased oral Epstein‐Barr virus DNA loads in patients with nasopharyngeal carcinoma in Southern China: A case‐control and a family‐based study

**DOI:** 10.1002/cam4.1597

**Published:** 2018-06-14

**Authors:** Wen‐Qiong Xue, Yong‐Qiao He, Xiao‐Yu Liao, Fang‐Fang Li, Ya‐Fei Xu, Feng‐Hua Xu, Xi‐Zhao Li, Qi‐Sheng Feng, Li‐Zhen Chen, Su‐Mei Cao, Qing Liu, Yi‐Xin Zeng, Wei‐Hua Jia

**Affiliations:** ^1^ State Key Laboratory of Oncology in South China Collaborative Innovation Center for Cancer Medicine Guangdong Key Laboratory of Nasopharyngeal Carcinoma Diagnosis and Therapy Sun Yat‐Sen University Cancer Center Guangzhou China; ^2^ Department of Endocrinology Xinqiao Hospital Third Military Medical University Chongqing China; ^3^ Department Cell Biology and Genetics Shenzhen University Health Science Center Shenzhen China; ^4^ School of Public Health Sun Yat‐Sen University Guangzhou China; ^5^ Cancer Center of Guangzhou Medical University Guangzhou China

**Keywords:** case‐control study, Epstein‐Barr virus, nasopharyngeal carcinoma, oral EBV DNA loads, Southern China

## Abstract

The link of nasopharyngeal carcinoma (NPC) with Epstein‐Barr virus (EBV) has been established for decades. Although an abnormal high level of EBV sero‐antibody spectrum and cell‐free circulating EBV DNA loads were exhibited in NPC patients, oral EBV DNA loads, which are primarily responsible for the EBV transmission, has not been previously studied in NPC patients. We conducted an epidemiological study to measure the oral EBV loads, viral components, and the relationship with the serum antibody titers in a large case‐control population (968 cases and 1656 controls) and a family‐based population (91 cases and 165 unaffected family members). EBV DNA loads were detected by quantitative PCR approach targeting the BamHI‐W region. Although a large individualized variation existed, we still observed a decreased oral EBV DNA loads in the population of NPC patients compared to that of healthy controls (ORs were 1.00, 0.69, 0.62, 0.33 classified by the quartiles of viral loads, *P*_trend_ < .001) and family members. In contrast, the elevated levels of oral EBV loads were present in asymptomatic males and elders, suggesting a different important source for EBV transmission. Notably, oral EBV loads were inversely associated with serum antibody titers of VCA‐IgA, EA‐IgA (All *P*_trend_ < .001) in the cases but not in the controls. Our study provides the first epidemiological data of oral EBV loads and viral components in NPC patients and controls in the highest risk area of Southern China, indicating that NPC status is unlikely to be an important determinant of EBV transmission.

## 1 INTRODUCTION

Epstein‐Barr virus (EBV) is a ubiquitous pathogen. It is a causal or contributing factor to a great variety of human disease, ranging from classic infectious mononucleosis (IM)^1^ to acute complications or extreme illness, such as encephalitis^2^ and several types of cancer.^1^, ^3^ In Southern China and Southeast Asia, nasopharyngeal carcinoma (NPC) has been identified as the highest associated cancer type with EBV.^4^, ^5^ The supporting evidences include the following: (1) the presence of EBV DNA or transcripts in almost all NPC cells,^6^, ^7^ but barely in normal epithelial cells^8^ (2) the detection of an abnormal high level of EBV sero‐antibody spectrum in NPC patients, including lytic‐related, or latent‐maintaining antibodies, such as anti‐VCA‐IgA, Zta‐IgA, EA‐IgA, and EBNA‐IgA,^9^, ^10^, ^11^ (3) the strong linkage between the aberrant high level of cell‐free circulating EBV DNA loads with the stage and prognosis of NPC.^12^, ^13^, ^14^ Recently, the circulating cancer‐derived EBV DNA in plasma has been established as a tumor marker for NPC and was used in screening for early asymptomatic nasopharyngeal carcinoma.^15^

Although EBV infectivity is significantly less than pathogens such as influenza, measles, chicken pox, and so on, intimate contact, mainly through the saliva or oropharyngeal secretions,^16^, ^17^, ^18^ could cause transmission of this virus.^19^ Despite the strong epidemiologic studies evidence that asymptomatic and mild symptomatic virus shedders are the major sources of infection in the community,^16^, ^20^ NPC patients, usually exhibits a high EBV serological profile, are considered, biasedly, with higher potential for disease transmission by people in general which results in considerable pressure and discrimination for NPC patients. Therefore, evaluating oral EBV loads in NPC cases and controls could possibly help to change this status, providing exact data on the oral viral loads of NPC patients in the endemic areas.

To date, only few studies have focused on the level of EBV loads in the site of primary EBV infection—oral cavity,^21^ and substantially lack of the systematic studies in the comparison of oral EBV load in NPC patients and normal controls. Here, we carry on an extensive epidemiological study to address these oral EBV loads‐related questions: (1) Is it possible that oral EBV DNA loads of NPC patients exhibit the consistent higher level as sero‐antibody or circulating EBV loads compared with the normal individuals? (2) What's the level of oral EBV loads of unaffected first‐degree relatives in NPC patients’ family? (3) Are there any fractions difference of oral EBV DNA between NPC patients and controls? In addition, we present the survey of oral EBV loads and its’ relationship with serum antibodies titers of anti‐VCA‐IgA and EA‐IgA in both the case‐control and family‐based studies. Our study provides the first epidemiological data of oral EBV loads in NPC patients in the highest risk area of Southern China.

## 2 MATERIALS AND METHODS

### 2.1 Study populations

A case‐control study, named as EPI‐NPC‐2005 project, was conducted during October 2005 and October 2007. In brief, cases were recruited from Sun Yat‐Sen University Cancer Center (SYSUCC), China. Eligible patients were requested to be lived in Guangdong for at least 5 years and have no previous diagnosis or treatment of NPC or other malignant neoplasm. Meanwhile, healthy controls were recruited from physical examination centers where the cases came from. The controls were strictly matched to the cases by age (±5 years), sex, geographic location. All the controls were Guangdong residents and have no history of cancer or immunologically mediated disease. A total of 1948 patients were included and 1845 patients (94.7%) completed the interviews. There are 418 patients refused to provide mouthwashes due to xerostomia and other reasons. In the same period, 2381 eligible controls were identified and 2275 (95.5%) interviews were finished. Sufficient mouthwash samples were collected from 1427 (77.3%) cases and 1656 (72.8%) controls. Data were collected via face‐to‐face interviews by trained interviewers. Samples were stored in 4°C before blood centrifuge and DNA extraction from mouthwash and then stored in −80°C for long period. Whole blood samples were also collected as previously reported.^22^ To avoid potential effects of radiotherapy or chemotherapy on oral EBV loads, we exclude 459 cases who have already received treatment. A total of 968 cases and 1656 controls were included in our final analysis. In addition to the case‐control population, we recruited 88 NPC pedigrees including 91 cases and 165 unaffected family members at SYSUCC during 2006‐2007. Three of the families have 2 NPC probands while the rest 88 families were with only 1 NPC patient. This research was approved by the ethical committee of SYSUCC, and informed consent was obtained from each subject.

### 2.2 Serological tests for VCA‐IgA and EA‐IgA antibodies

EBV VCA‐IgA and EA‐IgA antibody titers in serum were detected. Detailed methodological information has been described in our previous publications.^22^, ^23^ The VCA‐IgA and EA‐IgA assays were conducted using commercial kit (Zhongshan Bio‐tech Co Ltd, Zhongshan City, China, http://en.bio-kit.com/) following a standard immunoenzymatic procedure: (1) prepare B95‐8 cell smears and fix with acetone in the wells of slides; (2) add a series of diluted sera to the wells; (3) incubate the slides (30 minutes at 37°C) and wash them with phosphate buffered saline several times; (4) add peroxidase‐conjugated antihuman IgA antibody to the wells; (5) wash the slides with aminoethylcarbazole solution and H_2_O_2_ for 15 min. The slides were examined microscopically, and brown staining was reported positive. The technicians who detected these antibodies were blinded to the subjects’ case‐control status. All tests for the samples of cases and controls were conducted in the same laboratory and by the same technician.

### 2.3 Oral samples collection and DNA extraction

All the subjects were requested to not eat or drink for at least 30 minutes before sample collection. Mouthwash samples were collected by gargling with 10 mL physiological saline (0.9% NaCl) rinsing for 30 seconds at the date of first investigation. DNA was extracted by phenol‐chloroform within 12 hours after collection and stored in −80°C for subsequent experiment. Briefly, 500 μL of 10% SDS and 7 μL of 20 mg/mL RNase were added to 10 mL to each mouthwash sample. After 30‐minute incubation at 37°C, 20 μL of 20 mg/mL proteinase K was added, and the samples were incubated at 50°C for 30 minutes. Then, the equal volume of phenol: chloroform: isopentanol (25:24:1, pH 8.0) was added and centrifuge at 5000 rpm for 10 minutes at room temperature. The upper aqueous layer was carefully taken and extracted with equal volume of chloroform and 1/10 volume of 3 mol/L NaAc (pH 5.0) followed by addition of 2× volume of ice‐cold 100% ethanol. The precipitated DNA was pelleted, and then washed with 70% ethanol and dissolved in 50 μL nuclease‐free water. The samples of NPC case and control were processed following the identical procedures to avoid the potential biases caused during the sample collection and preparation.

To detect EBV DNA in different fractions of mouthwashes, additional NPC case and control samples, which are different from the samples used in the main evaluation, were collected. The samples were fractionated by centrifugation at 1500 *g* for 10 min, and the pellet was collected as peeled oral cells. The supernatant was filtered with 0.45 μm pore size filters and then ultra‐centrifuged at 60 000 *g* for 2 hours at 4°C to obtain the fragmented EBV DNA and cell‐free virions.^24^ DNA in these fractions were extracted by an automated workstation (Chemagic Star; Hamilton Robotic, Bonaduz, GR, Switzerland) using corresponding protocol.

### 2.4 Detection of EBV DNA copy number by quantitative real‐time PCR

A repetitive highly conserved BamHI‐W targets was used to quantify EBV DNA copy number by quantitative real‐time PCR.^25^, ^26^ EBV sequence was acquired from the GenBank database (accession number V01555). The qPCR system is consisted of the amplification primers: BW‐F, 5‐CCCAACACTCCACCACACC‐3; BW‐R, 5‐TCTTAGGAGCTGTCCGAGGG‐3; and a dual‐labeled fluorescent TaqMan probe: BW‐probe, 5‐(FAM)CACACACTACACACACCCACCCGTCTC(TAMRA)‐3. The probes were synthesized by Thermo Fisher Scientific (MA, USA) and have been reported in the previous study.^26^ The qPCR reactions were set up in a reaction volume of 8 μL, containing 4 μL Probes Master Mix, 0.8 μL primers (10 μmo/L), 0.2 μL probes (10 μmol/L), 2 μL template, and 1 μL nuclease‐free water. The qPCR reactions were initiated with predenaturation for 5 minutes at 95°C; followed by 45 cycles of denaturation for 30 seconds at 95°C, annealing for 30 seconds at 60°C, and extension for 15 seconds at 72°C. The qPCR was performed in 384‐well plate containing nuclease‐free water as negative control and standard samples as positive control. The standard ladders, which contained BamHI‐W region of the EBV genome (10^2^, 10^3^, 10^4^, 10^5^, 10^6^ and 10^7^ copies per 2 μL), were used to draw a standard curve by q‐PCR. The concentration of EBV DNA in mouthwashes (expressed as copy numbers per ml) was quantified using this standard curve. The samples of cases and control were tested in the same batch.

### 2.5 Statistical analyses

As the distribution of EBV DNA loads/mL was highly skewed, it was log10 transformed before analyses. The concentration of viral load was presented as median (M) and interquartile range (IQR). The comparisons of viral load were analyzed with Mann‐Whitney *U* test for 2 groups and Kruskal‐Wallis test for 3 or more groups. Logistic regression analysis was conducted to calculate the adjusted odds ratio (OR). EBV VCA‐IgA titers greater than or equal to 1:40 or EA‐IgA titers at least 1:10 were taken as positive. The adjustment factors include sex, age, education, smoking, intake of salted fish and fresh fruit. All statistical tests were 2‐sided and considered significant as *P *<* *.05. Analyses were performed in STATA 10.0 (Stata Corp, College Station, TX).

## 3 RESULTS

### 3.1 Demographic characteristics of the study populations

The present case‐control study included 968 cases and 1656 controls. The average age for both the cases and controls was 46 years. About 70% of the subjects were males in both cases and controls group. The distribution of sex and age were comparable between cases and controls. The education level was higher in healthy controls than in cases, so it was fully adjusted in the further analysis (Table 1). The family‐based study included 91 NPC patients and 165 unaffected members from 88 families. Most of the family members (N = 108) were first‐degree relatives of the patients, including parents, offspring, and siblings. Thirty were their spouses, and 27 were other relatives like cousins or nephews.

**Table 1:** The associations between demographic, clinical characteristics, and nasopharyngeal carcinoma in Guangdong population

Variables^a^	No. of Healthy controls (%)	No. of NPC cases (%)	Adjusted OR (95%CI)^b^	*P* c
Age	46 ± 12	46 ± 11		.556
Sex				
Female	491 (29.65)	254 (26.24)		
Male	1165 (70.35)	714 (73.76)		.062
Education years				
≤6	225 (13.63)	237 (24.56)	1.00 (reference)	/
7‐12	891 (53.97)	604 (62.59)	0.57 (0.45‐0.71)	<.001
>13	535 (32.40)	124 (12.85)	0.20 (0.14‐0.26)	<.001
*P*_trend_ d				<.001
VCA‐IgA				
Negative	1322 (81.00)	37 (3.85)	1.00 (reference)	/
Positive	310 (19.00)	923 (96.15)	115.08 (79.16‐167.29)	<.001
EA‐IgA				
Negative	1628 (99.75)	215 (22.42)	1.00 (reference)	/
Positive	4 (0.25)	744 (77.58)	1409.70 (518.80‐3830.53)	<.001
Oral EBV load				
P_0_‐P_25_	412 (24.88)	366 (37.81)	1.00 (reference)	/
P_25_‐P_25_	414 (25.00)	251 (25.93)	0.69 (0.55‐0.86)	.001
P_50_‐P_75_	414 (25.00)	218 (22.52)	0.62 (0.49‐0.79)	<.001
P_75_‐P_100_	416 (25.12)	133 (13.74)	0.33 (0.26‐0.43)	<.001
*P*_trend_ d				<.001

a For age, mean, and standard deviation were descried among cases and controls.

b Multivariable logistic regressions were used by adjusting age (continuous variables), sex (males and females), education years (≤6, 7‐12, >13), cigarette smoking pack‐years (non‐smoker, <20, ≥20), alcohol drinking (non‐drinker, ≤1 drink per day, >1 drink per day), consumption of preserved vegetable (less than monthly, monthly, weekly, or more), and consumption of salted‐fish (less than monthly, monthly, weekly, or more).

c Mann‐Whitney *U* test were used for comparison of age between 2 groups; chi‐square test was used for comparison of sex between 2 groups; multivariable logistic regressions were used for comparison of other category variables between 2 groups.

d Linear trends tests were performed by treating ordered categorical variables as continuous variables.

### 3.2 Analytical sensitivity and reproducibility

Limits of detection (LOD) of the quantitative real‐time PCR assays were determined with serial dilutions of control plasmids. As showed in Figure S1, a robust LOD of ≤5 copies/reaction was detected for BamHI‐W targeted qPCR. The assay reproducibility was further studied by duplicated analysis of quantification standards or NPC case and control samples. The assay variation was calculated with measure quantities of target DNA. The intraclass correlation coefficient (ICC) for the duplicated samples of NPC cases, controls, and standards were 88.84%, 82.04%, and 99.63%, respectively.

### 3.3 Comparison of oral EBV DNA loads between NPC cases and healthy controls

In this case‐control population, oral EBV DNA loads were compared between cases and controls. Unexpectedly, we did not observe an enhanced positive rate of oral EBV DNA in cases compared with healthy controls. On the contrary, we found a decreased positive rate of 66.8% for oral EBV detection in NPC patients compared with that of 80.7% in healthy controls (*P *<* *.001), and a decreased median viral load (log10 copies/mL) of 3.97 (IQR: 0‐4.92) in cases compared with controls of 4.53 (IQR: 3.04‐5.45) (Table 2). Interestingly, high levels of EBV loads were associated with males and elders in both case and control group. The median loads for males and females were 4.06 vs 3.53 (*P *=* *.015) in cases and 4.72 vs 3.85 (*P *<* *.001) in controls. Moreover, the loads increased with age in both case and control group (*P*_trend_ < .05). There was a marginal significant association between EBV loads and education levels in NPC patients (*P*_trend_ = .042), while not in controls **(** *P*_trend_ = .606, Table 2).

**Table 2:** Comparison of oral EBV DNA Loads between NPC cases and Healthy Controls by subgroup analysis

Variable	NPC cases			Healthy controls			*P* c
	N (%)	Median^a^	Median (IQR)^b^	N (%)	Median^a^	Median (IQR)^b^	
Total	968	9309	3.97 (0.00‐4.92)	1656	33 978	4.53 (3.04‐5.45)	<.001
Age							
<40	265 (27.38)	6128	3.79 (0.00‐4.77)	511 (30.86)	19 776	4.30 (2.39‐5.21)	<.001
40‐49	318 (32.85)	7561	3.88 (0.00‐4.89)	500 (30.19)	19 490	4.30 (2.94‐5.39)	<.001
50‐59	269 (27.79)	13 613	4.13 (0.00‐4.96)	420 (25.36)	66 577	4.82 (3.48‐5.74)	<.001
≥60	116 (11.98)	24 384	4.39 (2.62‐5.17)	225 (13.59)	58 857	4.77 (3.54‐5.65)	.004
*P* c			.028			<.001	
Sex							
Female	254 (26.24)	3379	3.53 (0.00‐4.88)	491 (29.65)	7125	3.85 (0.00‐5.01)	.010
Male	714 (73.76)	11 513	4.06 (0.00‐4.93)	1165 (70.35)	52 231	4.72 (3.34‐5.64)	<.001
*P* c			.015			<.001	
Education							
≤6	237 (24.56)	4178	3.62 (0.00‐4.72)	225 (13.63)	26 908	4.43 (2.89‐5.45)	<.001
7‐12	604 (62.59)	12 505	4.10 (0.00‐4.99)	891 (53.97)	35 403	4.54 (3.04‐5.47)	<.001
>13	124 (12.85)	8519	3.93 (0.00‐4.96)	535 (32.40)	36 091	4.56 (3.07‐5.41)	.001
*P* c			.042			.606	

a Median represents the median value of the oral EBV load copy number in per milliliter mouth washing.

b Median (IQR) represents the log10 transformed median value and interquartile value of oral EBV load copy number in per milliliter mouth washing.

c Mann‐Whitney *U* tests were used for comparison of oral EBV load between 2 groups and Kruskal‐Wallis tests were used for comparisons of oral EBV load among 3 or more groups.

In addition, as shown in Table 1, an association of EBV loads with NPC after adjusting for age, sex, and education, consumption of salted fish, fresh fruit, and smoking was determined by using multivariate logistic regression analysis. ORs were 1.00, 0.69, 0.62, 0.33 in subgroups of P_0_‐P_25,_ P_25_‐P_50,_ P_50_‐P_75,_ P_75_‐P_100,_ respectively (*P*_trend_ < .001), when we stratified the individuals into 4 subgroups based on quartiles of viral loads in healthy controls. Interestingly, an adverse association existed between EBV loads and NPC.

### 3.4 Comparison of oral EBV DNA loads in NPC cases, unaffected family members, and healthy controls

To investigate whether family members of NPC patients could carry a changed oral EBV loads, we recruited 165 unaffected members and 91 NPC patients in 88 families, among them, 3 families have more than 1 patient. Consistent with case‐control study, the cases in families exhibited lower median loads of 3.65 than those of unaffected relatives of 4.54 (*P *=* *.040) and healthy controls of 4.53 (*P *=* *.004) (Table 3). Among the NPC families, the median loads in the parents, siblings, and spouses were 4.97, 4.85, and 4.39, respectively. All these unaffected relatives had higher oral EBV loads than the probands (*P *=* *.010, .030, .038 for the parents, siblings, and spouses, respectively) and were not significant different from the former controls (*P *>* *.05). We found EBV loads of offspring showed a lower median loads level of 3.31, which could be explained by a predominantly distribution of younger age in this group. No significant difference was observed between NPC probands in these families and the cases from EPI‐NPC‐2005 project (*P *=* *.502).

**Table 3:** Comparison of Oral EBV DNA loads in NPC pedigrees and general controls

Subjects	EBV loads (copies/mL)^a^		Median^b^	Median (IQR)^c^	*P* d
	<33 978, N(%)	≥33 978, N(%)			
Healthy controls	828 (50.00)	828 (50.00)	33 978	4.53 (3.04‐4.53)	Ref.
Unaffected relatives	82 (49.70)	83 (50.30)	34 930	4.54 (2.88‐5.68)	.719
Parents	9 (37.50)	15 (62.50)	100 352	4.97 (3.13‐6.07)	.142
Offspring	27 (60.00)	18 (40.00)	2064	3.31 (2.66‐5.72)	.266
Sibling	17 (43.59)	22 (56.41)	71 519	4.85 (2.94‐5.43)	.456
Spouse	16 (53.33)	14 (46.67)	25 269	4.39 (3.07‐5.50)	.590
Other relatives	13 (50.00)	14 (50.00)	24 292	3.93 (2.43‐5.62)	.556
NPC patients	65 (71.43)	26 (28.57)	4464	3.65 (2.85‐4.63)	.004
					.040^e^

a Oral EBV loads were assigned into 2 groups based on the median of that in healthy controls (33 978 copies/mL).

b Median represents the median value of the oral EBV load copy number in per milliliter mouth washing.

c Median (IQR) represents the log10 transformed median value and interquartile value of oral EBV load copy number in per milliliter mouth washing.

d Mann‐Whitney *U* test was used for comparison of oral EBV load between general healthy subjects and NPC pedigree members.

e Mann‐Whitney *U* test was used for comparison of oral EBV load between unaffected family members and NPC probands.

### 3.5 Component analysis of EBV DNA in the fractions of mouthwashes

**Figure 1:**
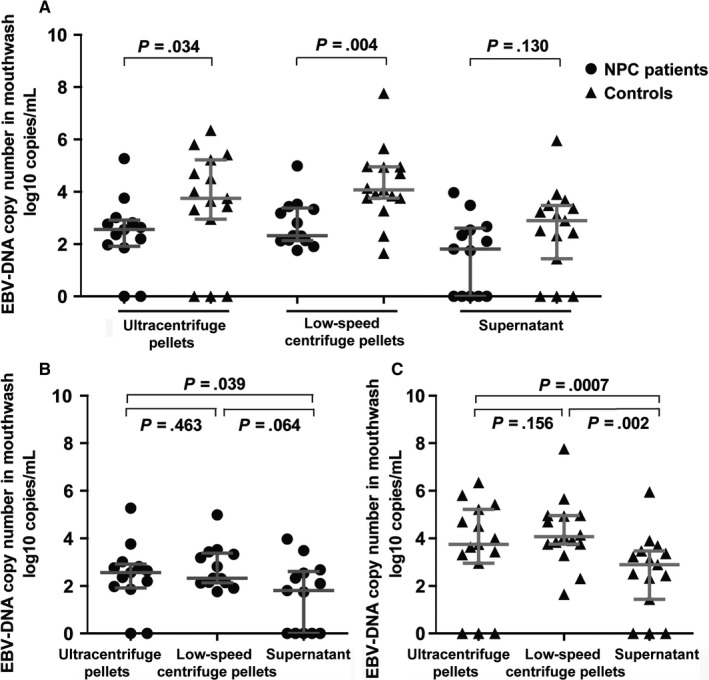
EBV DNA loads in different fractions of mouthwashes in NPC patients and controls. A, Comparison of EBV DNA loads in the 3 fractions of mouthwashes between NPC patients and controls. B, Comparisons of EBV DNA loads among the 3 fractions of mouthwashes in NPC patients. C, Comparisons of EBV DNA loads among the 3 fractions of mouthwashes in controls. The horizontal lines represented the medians and quartiles of EBV DNA loads for each fraction of mouthwashes in patients and controls. The vertical line represented the corresponding interquartile range. The comparison of viral load between NPC patients and controls was performed using Mann‐Whitney *U* test.

To further investigate how EBV was presented in mouthwashes, we fractionated mouthwash samples by low and ultra‐speed centrifugation.^24^ The pellets from ultra‐speed centrifugation and low‐speed centrifugation were expected to contain cell‐free virions and cell‐derived viruses, respectively. The supernatant from ultra‐speed centrifugation may contain fragmented EBV DNA. EBV DNA loads were detected separately in these 3 components. In results, EBV DNA could be detected in all of 3 fractions (Figure 1). Consistent with the results from previous case‐control and family‐based studies, EBV DNA loads kept on remaining a lower level in patients compared with those in controls in each components of pellets from ultra‐speed centrifugation (Figure 1A, lane 1), pellets from low‐speed centrifugation (Figure 1A, lane 2) and supernatant (Figure 1A, lane 3), the median loads were 2.56 vs 3.74 (*P *=* *.034) and 2.32 vs 4.06 (*P *=* *.004), respectively, in former 2 forms (Figure 1A, lane 1 and 2), the difference was not significant for viral DNA from supernatant (lane 3). Moreover, we found that EBV DNA loads of pellets from ultra and low‐speed centrifugation, which contain cell‐free virions and peeled oral cell‐derived viruses, were more abundant in mouthwashes compared with the viral loads from supernatant (Figure 1B,C); however, due to the limited sample size, this trend needs to be confirmed in the future.

### 3.6 The relationship between oral viral DNA loads and clinical characteristics

Serum EBV antibody such as anti‐VCA‐IgA and EA‐IgA were aberrantly elevated in NPC patients, but a decreased trend of EBV DNA loads in oral cavity was observed in this study. Interestingly, statistical analysis indicates that oral EBV loads were indeed inversely associated with serum antibody titers of VCA‐IgA and EA‐IgA (*P*_trend_ < .001), but this adverse relationship was only observed in cases not in controls (Table 4). In detail, when we use individuals of VCA‐IgA ≤ 1:40 as reference, the ORs for the higher titers groups (1:80‐1:160, 1:320‐1:640, >1:640) were 0.51, 0.38, and 0.28 in the comparison of high and low oral EBV loads groups, respectively. Similarly, the corresponding ORs for EA‐IgA antibody were 0.63, 0.52, and 0.14 in different titers groups (1:20‐1:40, 1:80‐1:160, >1:160), respectively. Dose‐response pattern existed between EBV antibodies and oral viral loads (Table 4). Noteworthy, we did not observe any relationship between the antibodies and oral viral loads in healthy control group (Table 4). In brief, we firstly observed that patients who have elevated antibody titers linked to decreased oral EBV DNA loads.

**Table 4:** The associations between potential risk factors and oral Epstein‐Barr virus load levels among NPC cases and healthy controls

Variables	NPC cases				Healthy controls			
	EBV loads (copies/mL)^a^		Adjusted OR (95% CI)^b^	*P*	EBV loads (copies/mL)^a^		Adjusted OR (95% CI)^b^	*P*
	<33 978 , N(%)	≥33 978, N(%)			<33 987, N(%)	≥33 987, N(%)		
Age								
<40	182 (29.50)	83 (23.65)	1.00 (reference)	/	288 (34.78)	223 (26.93)	1.00 (reference)	/
40‐49	206 (33.39)	112 (31.91)	1.36 (0.94‐1.96)	.101	271 (32.73)	229 (27.66)	1.06 (0.82‐1.38)	.647
50‐59	164 (26.58)	105 (29.91)	1.73 (1.18‐2.55)	.005	173 (20.89)	247 (29.83)	1.75 (1.32‐2.34)	<.001
≥60	65 (10.53)	51 (14.53)	2.18 (1.34‐3.56)	.002	96 (11.59)	129 (15.58)	1.65 (1.17‐2.34)	.005
*P*_trend_ c				<.001				<.001
Sex								
Female	172 (27.88)	82 (23.36)	1.00 (reference)	/	308 (37.20)	183 (22.10)	1.00 (reference)	/
Male	445 (72.12)	269 (76.64)	1.34 (0.90‐2.00)	.155	520 (62.80)	645 (77.90)	1.65 (1.25‐2.18)	<.001
Education years								
≤6	166 (26.99)	71 (20.29)	1.00 (reference)	/	118 (14.29)	107 (12.97)	1.00 (reference)	/
7‐12	374 (60.81)	230 (65.71)	1.55 (1.09‐2.21)	.015	444 (53.75)	447 (54.18)	1.29 (0.94‐1.78)	.111
>13	75 (12.20)	49 (14.00)	1.65 (1.00‐2.72)	.049	264 (31.96)	271 (32.85)	1.59 (1.11‐2.26)	.011
*P*_trend_ c				.027				.010
VCA‐IgA								
≤1:40	64 (10.37)	73 (20.80)	1.00 (reference)	/	822 (99.28)	821 (99.15)	1.00 (reference)	/
1:40‐1:160	199 (32.25)	120 (34.19)	0.51 (0.34‐0.78)	.002	5 (0.60)	5 (0.60)	0.95 (0.26‐3.51)	.934
1:160‐1:640	288 (46.68)	134 (38.18)	0.38 (0.25‐0.57)	<.001	1 (0.12)	2 (0.24)	1.47 (0.13‐17.09)	.759
>1:640	66 (10.70)	24 (6.84)	0.28 (0.15‐0.51)	<.001	0 (0.00)	0 (0.00)		.862
*P*_trend_ c				<.001				
EA‐IgA								
≤1:10	224 (36.30)	166 (47.29)	1.00 (reference)	/	828 (100.00)	828 (100.00)	1.00 (reference)	/
1:10‐1:40	218 (35.33)	114 (32.48)	0.63 (0.46‐0.87)	.005	0 (0.00)	0 (0.00)	/	/
1:80‐1:160	149 (24.15)	68 (19.37)	0.52 (0.36‐0.75)	<.001	0 (0.00)	0 (0.00)	/	/
>1:160	26 (4.21)	3 (0.85)	0.14 (0.04‐0.49)	.002	0 (0.00)	0 (0.00)	/	/
*P*_trend_ c				<.001				/
Overall stage								
I	19 (3.34)	12 (3.55)	1.00 (reference)	/				
II	93 (16.34)	56 (16.57)	1.10 (0.48‐2.49)	.824				
III	304 (53.43)	168 (49.70)	0.98 (0.46‐2.13)	.968				
IV	153 (26.89)	102 (30.18)	1.11 (0.50‐2.46)	.800				
*P*_trend_ c				.840				
T stage								
T1	39 (6.63)	15 (4.36)	1.00 (reference)	/				
T2	163 (27.72)	77 (22.38)	1.30 (0.66‐2.53)	.446				
T3	276 (46.94)	158 (45.93)	1.58 (0.83‐3.00)	.165				
T4	110 (18.71)	94 (27.33)	2.32 (1.17‐4.57)	.015				
*P*_trend_ c				.002				
N stage								
N0	118 (20.03)	108 (31.40)	1.00 (reference)	/				
N1	224 (38.03)	146 (42.44)	0.74 (0.52‐1.04)	.085				
N2	192 (32.60)	73 (21.22)	0.40 (0.27‐0.59)	<.001				
N3	55 (9.34)	17 (4.94)	0.32 (0.17‐0.60)	<.001				
*P*_trend_ c				<.001				
M								
M0	575 (97.79)	333 (97.65)	1.00 (reference)	/				
M1	13 (2.21)	8 (2.35)	0.80 (0.29‐2.16)	.653				

a Oral EBV loads were assigned into 2 groups based on the median of that in healthy controls (33 978 copies/mL).

b Multivariable logistic regressions were used by adjusting age (continuous variables), sex (males and females), education years (≤6, 7‐12, >13), cigarette smoking pack‐years (nonsmoker, <20, ≥20), alcohol drinking (non‐drinker, ≤1 drink per day, >1 drink per day), consumption of preserved vegetable (less than monthly, monthly, weekly, or more), and consumption of salted‐fish (less than monthly, monthly, weekly, or more).

c Linear trends tests were performed by treating ordered categorical variables as continuous variables.

To assess the possible relationship between clinical stages and oral EBV load, we further grouped the patients by clinical stage at diagnosis. As showed in Table 4, higher oral EBV loads was not associated with overall NPC stages. However, T4 stage was significantly associated with higher oral EBV loads (OR = 2.32, 95% CI = 1.17‐4.57, *P *=* *.015). On the contrary, N stage was inversely associated with high oral EBV loads, the corresponding ORs for N2 and N3 groups were 0.40 (95% CI = 0.27‐0.59, *P* < .001) and 0.32 (95% CI = 0.17‐0.60, *P* < .001), respectively. No association was observed between M stage and oral EBV loads (Table 4).

## 4 DISCUSSION

EBV has long been established as a causal agent for serious diseases including NPC. The viruses usually transmitted by close contacts involving saliva exchange like deep, open‐mouth kissing. After primary infection, the viruses may replicate in oral epithelial cells and released into saliva, which can lead to the infection of new hosts. In southern China and other high‐risk area of NPC, mounting evidence has confirmed that the EBV in NPC patients represents a more “active” status,^27^ which leads to the misconception that NPC patients are possibly with higher potential for disease transmission compared to healthy population. This results in a tremendous social discrimination against NPC patients and their family members in NPC endemic area. Surprisingly, oral EBV load has not been paid more attention in NPC patients and controls. Therefore, illustrating oral EBV loads and virial components in NPC patients, their unaffected family members, and general population could help to dissolve the longstanding public health question.

To our knowledge, this was the first study systematically evaluating oral EBV loads in NPC patients, their family members as well as in common healthy people. We carried out a large case‐control study, followed by a family‐based study and a detailed oral viral fraction analysis. We revealed that although NPC patients exhibited an elevated sero‐antibody against EBV, their oral EBV DNA loads level and cell‐free EBV virions were significantly decreased compared with general population, implying that NPC patients were not the primary source of EBV infection in high‐risk area of NPC. The family study showed the similar results, confirming that intimate contacts with NPC patients would not elevate the risk of EBV infection.

Our research included a case‐control population (EPI‐NPC‐2005 project) which has been reported in a series of studies regarding EBV, environmental and genetic factors on NPC etiology previously.^23^, ^28^, ^29^ To exclude potential effect of clinical treatment on the level of oral EBV loads, the mouthwashes from patients who have already initiated their treatment were excluded. Followed by case‐control study, oral viral load in patients and their unaffected familial members were assessed. Oral viral load of relatives which contacted closely with NPC patients can reflect the burden of EBV infections transmitted from NPC patients. As showed clearly in this study, the burden of EBV infection was not higher in NPC family members than healthy controls. In summary, this study provided the robust evidences that NPC patients are not the main culprit of EBV infection in high‐risk area of NPC, which is against the conventional view especially in those low socioeconomic populations that NPC patients are more “contagious” in endemic areas in China.

It is worth noting that the oral EBV loads variability due to the dynamics of virus shedding^17^ or detection methods^30^ should be carefully considered when comparing the oral EBV loads in NPC cases and controls. As showed in recent report by Hadinoto,^17^ EBV shedding into saliva is relatively stable over short period but highly variable over longer period within individual. Unfortunately, for this was a cross‐sectional designed study, we cannot observe the variability of the population‐based EBV DNA loads over time. Nevertheless, a well‐designed epidemiologic study with larger sample size is required to see the EBV DNA loads of population level in addition to individual level. In our results, despite a large individualized variation existed, we still observed a decreased oral EBV DNA loads in population level in NPC patients compared to healthy controls.

In this study, we found that oral EBV loads in NPC patients were not only lower than that in healthy individuals; but reversely associated with anti‐VCA‐IgA and anti‐EA‐IgA, 2 antibodies against EBV lytic period, suggesting a simultaneous emergence of enhanced innate immunity response to EBV lytic period and reduced local EBV activation in oral cavity. EBV is difficult to infect normal nasopharynx epithelial cells, but can infect almost all NPC tumor tissues and maintained in a latent state in NPC tumor cells.^6^, ^31^ EBV shedding in oral cavity EBV lytic cycle may be suppressed in EBV‐associated epithelial carcinomas.^32^ We proposed that EBV reproductive activity in oral cavity may be suppressed due to an abnormal switch between lytic and latent period in the pathogenesis of NPC. Previous study also suggested that some microenvironment signals may play roles in the suppression of EBV reproduction in oral cavity.^33^, ^34^ A recent study found that membrane vesicles secreted from oral epithelial cells of healthy persons harbored mir‐200 family members, which can favor lytic replication of EBV,^34^ while these miRNAs were found down‐regulated in nasopharyngeal carcinoma samples.^33^ The adverse relationship of oral EBV loads and serum antibody titers, we observed here, may closely link to NPC development. Indeed, the inverse relationship between oral EBV loads and N stage was in concert with the results of the serum antibody levels, but differently, T4 stage was found to be associated with higher oral EBV loads. Previous studies revealed aberrant levels of EBV antibodies, including potential neutralized antibody, can be detected in saliva of NPC patients while not in healthy EBV carriers.^35^, ^36^ A newly published paper reported that NA1‐expressed cells, antigen presenting cells (APCs), B cells can be all detected in local tumor environment of NPC.^37^ Thus, the oral EBV loads may be influenced by both local tumor and immune microenvironment in oral cavity. There was no obviously association between serum EBV antibodies and oral viral loads in health controls. However, this lack of association may be due to small sample size, as only a small fraction of healthy people were seropositive for EBV.

In addition, our data revealed that asymptomatic people, especially older people (≥50 years) and males, have higher oral EBV loads; thus, they could be the potential source of EBV transmission, leading to EBV reinfection or early primary infection. Previous studies have reported that early primary EBV infection may be attributed to low hygiene level and crowed living situation.^38^, ^39^ Although rapid economic development has improved the living condition and hygiene standard in China, traditional Chinese family pattern are still complicated and crowded. The family members eat together and are more likely to share chopsticks. These behaviors may result in EBV transmission in family members and early primary infection in preadolescent children, which may increase the potential of EBV‐related diseases, like periodontitis, or even hypothesize to increase potential of other EBV‐related malignancy.^31^, ^40^ Therefore, to block EBV infection and reduce the burden of EBV‐related diseases in this area, it is advocated to reduce the high‐risk behaviors involving saliva exchange like adults prechewing food.

We need to point out that, the oral EBV DNA loads may be affected by collection techniques. However, for each subject, the mouthwash was restricted to 10 mL and swirled for 30 seconds. In addition, we detected copy numbers of host β‐globin gene in 890 NPC cases and 650 controls, in the same batch of PCR targeting EBV BamHI‐W region. However, the copy numbers of β‐globin gene were significantly higher in cases than controls (log10 transformed, median: 6.38, vs 5.81, *P *<* *.001). The oral EBV loads of NPC patients could only be overestimated. Therefore, the tendency of lower EBV loads in NPC patients is reliable, not modified by the potential disparity of total DNA amount between cases and controls.

To summarize, this first systematic oral EBV load study indicate that asymptomatic people are more important sources for EBV infection than NPC patients. Moreover, in the healthy people, elders and males tend to have higher EBV load. These findings will better replenish the current knowledge on EBV infection and help to dispel discrimination against NPC patients in the high‐endemic areas.

## CONFLICT OF INTEREST

The authors declare that they have no conflict of interest to this work.
