# Supplementary figures and images for: Decreased oral Epstein‐Barr virus DNA loads in patients with nasopharyngeal carcinoma in Southern China: A case‐control and a family‐based study
Source: Cancer Med. 2018 Jun 14;7(7):3453–64. doi: 10.1002/cam4.1597 (PMC6051183; doi:10.1002/cam4.1597)

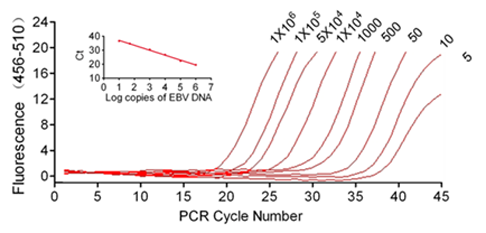

Supplement: Supplementary file 1 [file CAM4-7-3453-s001.tif]
