# Supplementary material for: Decreased oral Epstein‐Barr virus DNA loads in patients with nasopharyngeal carcinoma in Southern China: A case‐control and a family‐based study
Source: Cancer Med. 2018 Jun 14;7(7):3453–64. doi: 10.1002/cam4.1597 (PMC6051183; doi:10.1002/cam4.1597)
Supplement: Supplementary file 2 [file CAM4-7-3453-s002.docx]

**Supplemental Figure1. Real-time quantitative PCR assay targeting the BamHI-W region of EBV DNA.**

The results of serial standard DNA dilutions containing known quantities of EBV DNA were subjected to real-time quantitative PCR analysis of the BamHI-W region. The PCR system was sensitive enough to detect 5 copies per reaction. The inset shows a plot of the threshold cycle (Cγ) against the target quantity (Log). The EBV DNA content of each sample was derived by interpolation of the mean Cγ value of the triplicate assays with the standard curve.
